# **RETRACTION:** Frequent Alterations of the Candidate Genes hMLH1, ITGA9 and RBSP3 in Early Dysplastic Lesions of Head and Neck: Clinical and Prognostic Significance

**DOI:** 10.1111/cas.70372

**Published:** 2026-03-19

## Abstract

**RETRACTION**: GhoshA. , GhoshS. , MaitiG.P. , SabbirM.G. , ZabarovskyE.R. , RoyA. , RoychoudhuryS. , and PandaC.K. , “Frequent Alterations of the Candidate Genes hMLH1, ITGA9 and RBSP3 in Early Dysplastic Lesions of Head and Neck: Clinical and Prognostic Significance,” Cancer Science 101, no. 6 (2010): 1511‐1520, 10.1111/j.1349-7006.2010.01551.x.20412120 PMC11159363

The above article, published online on 19 May 2010 in Wiley Online Library (http://onlinelibrary.wiley.com/), has been retracted by agreement between the journal Editor‐in‐Chief, Masanori Hatakeyama; the Japanese Cancer Association; and John Wiley & Sons Australia, Ltd. A third party contacted the journal with concerns about duplicated images in the figures. Following an investigation, multiple duplications were found throughout Figures 2A, 3B, 4B, and supporting information Figure 2. Additionally, portions of some figures were duplicated in later articles by some of the same authors: Figure 2A in Dasgupta et al. 2016 (https://doi.org/10.2217/fon‐2016‐0289); Figures 2A and 3A in Ghosh et al. 2011 (https://doi.org/10.1245/s10434‐011‐1991‐x); and Figure 3A in Bhattacharya et al. 2013 (https://doi.org/10.1245/s10434‐012‐2715‐6). Due to the scope of the errors, the editor has lost confidence in the results reported, and therefore the article must be retracted. The authors have been informed of this decision.

**RETRACTION**: A. Ghosh , S. Ghosh , G.P. Maiti , M.G. Sabbir , E.R. Zabarovsky , A. Roy , S. Roychoudhury , and C.K. Panda , “Frequent Alterations of the Candidate Genes hMLH1, ITGA9 and RBSP3 in Early Dysplastic Lesions of Head and Neck: Clinical and Prognostic Significance,” Cancer Science 101, no. 6 (2010): 1511‐1520, 10.1111/j.1349-7006.2010.01551.x.20412120 PMC11159363

The above article, published online on 19 May 2010 in Wiley Online Library (http://onlinelibrary.wiley.com/), has been retracted by agreement between the journal Editor‐in‐Chief, Masanori Hatakeyama; the Japanese Cancer Association; and John Wiley & Sons Australia, Ltd. A third party contacted the journal with concerns about duplicated images in the figures. Following an investigation, multiple duplications were found throughout Figures 2A, 3B, 4B, and supporting information Figure 2. Additionally, portions of some figures were duplicated in later articles by some of the same authors: Figure 2A in Dasgupta et al. 2016 (https://doi.org/10.2217/fon‐2016‐0289); Figures 2A and 3A in Ghosh et al. 2011 (https://doi.org/10.1245/s10434‐011‐1991‐x); and Figure 3A in Bhattacharya et al. 2013 (https://doi.org/10.1245/s10434‐012‐2715‐6). Due to the scope of the errors, the editor has lost confidence in the results reported, and therefore the article must be retracted. The authors have been informed of this decision.

